# Differential transcriptomic responses in carp cell lines following activation of antiviral immune responses by poly I:C

**DOI:** 10.1016/j.cirep.2026.200297

**Published:** 2026-06-29

**Authors:** Laurie Smeaton, Richard Paley, Irene Cano Cejas, Jason W. Holland, Samuel A.M. Martin

**Affiliations:** a Scottish Fish Immunology Research Centre, School of Biological Sciences, University of Aberdeen, Aberdeen, AB24 2TZ, UK; b Cefas Weymouth Laboratory, Barrack Road, The Nothe, Weymouth, Dorset, DT4 8UB, UK

**Keywords:** Common carp, poly I:C, cell lines, antiviral, gene expression

## Abstract

Common Carp Brain (CCB) and Koi Fin-1 (KF1) are two cell lines derived from common carp (*Cyprinus carpio*) that are utilised in the characterisation of common carp viruses. Cyprinid Herpesvirus-3 (CyHV-3) and Carp Edema Virus (CEV) are key global concerns for the common carp farming industry owing to their rapid spread, high fish mortalities and associated economic impacts. This highlights the importance of utilising cell lines for early diagnosis, viral characterisation, uncovering host antiviral responses, and development of therapeutic approaches, and underlines the importance of cell line choice. To examine the antiviral / interferon response of the CCB and KF1 cell lines, cells were stimulated with the viral dsRNA mimetic, poly I:C for 24 hours. RNA-Seq analysis was undertaken to uncover transcriptome-wide antiviral responses in each cell line to help further understand their research application potential. Cell line specific antiviral signatures were detected with more pronounced upregulation of antiviral genes (eg. ISG15, RIGI, IRF3 and IRF7) and higher basal expression of the classical dsRNA receptor, Toll-like Receptor 3 (TLR3) in CCB compared to KF1. Overall, this study has uncovered a marked poly I:C mediated antiviral immune response in the CCB cell line, contrasting with a much less pronounced antiviral response in the KF1 cell line.

## Highlights

• CCB and KF1 respond differently to poly I:C.
• A clear type-I interferon response was seen in both cell lines.
• CCB exhibited a more pronounced antiviral response than KF1.
• Magnitude of antiviral gene expression was higher in CCB than KF1.

## Introduction

Aquaculture is a rapidly growing industry that provides protein for human consumption globally [1], with 87.5 million tonnes of aquatic animals produced in 2020 [24]. As a key global aquaculture species with over 4.1 million tonnes produced globally in 2021, common carp (*Cyprinus carpio*; herein denoted as carp) is important for global food security [25]. Disease challenges affect production and welfare of carp, with viral diseases being key global concerns for carp aquaculture due to viruses such as Cyprinid Herpesvirus-3 (CyHV-3), Carp Edema Virus (CEV), and Spring Viraemia of Carp Virus (SVCV) [2,51,64].

Two key cell lines used in the diagnosis and research of carp viruses are Common Carp Brain (CCB) [67] and Koi Fin-1 (KF1) [33]. Both are used in virological research and diagnosis, as well as for assessment of carp immunological parameters, especially in relation to viral infections [1,10,81]. Two principal viral pathogens that result in economic losses are CyHV-3 and CEV, the etiological agents of Koi Herpesvirus Disease (KHVD) and Koi Sleepy Disease (KSD) respectively [30,51]. CyHV-3 can be propagated in several cell lines, including non-*C. carpio* cell lines, such as Tol/FL and AU cell lines derived from silver carp (*Hypophthalmichthys molitrix*) and goldfish (*Carassius auratus*) fin tissues respectively [16]. However, it is more commonly cultured in the CCB and KF1 cell lines [64]. In contrast, CEV has currently not been successfully cultured within any cell lines, hindering diagnostics, vaccine development, and characterisation of the viral agent [62].

Detection of viral pathogens occurs through recognition of pathogen-associated molecular patterns (PAMPs) by cell-located Pattern Recognition Receptors (PRRs) [77]. A key receptor in vertebrate host viral detection is toll-like receptor 3 (TLR-3). Which specifically binds to and is activated by dsRNA. This leads to the activation of the antiviral signalling cascade, characterised by cytokine release, particularly type-1 interferons (IFNs) [7]. Released IFNs bind to IFN receptors, leading to the activation of TYK2 and JAK1 and phosphorylation of STAT1 and STAT2 transcription factors. This drives the dimerization of Interferon Regulatory Factor 9 (IRF9) and formation of the Interferon Stimulated Gene Factor 3 (ISGF3) complex [71,94]. ISGF3 undergoes nuclear translocation binding to Interferon-stimulated response element (ISRE) motifs within the promoters of interferon-stimulated genes (ISGs). This leads to the downstream expression of a diverse range of antiviral mechanisms [43,50].

In contrast to terrestrial vertebrates, additional whole genome duplication events (WGD) in fish, especially carp, have significantly increased the complexity of fish antiviral responses owing to the presence of duplicated immune genes. This warrants functional genomic approaches to fully understand the nature of fish-virus interactions and to begin uncovering the role of paralogous antiviral immune genes. WGD events can result in sub- or neofunctionalization of retained genes [48]. Teleosts have undergone three WGDs, with two ancestral WGDs, including a teleost-specific WGD occurring at the root of the teleost lineage around 320∼350 MYA [29]. Common carp have undergone a total of four WGD events and are an example of an allotetraploid species arising from a hybridisation event that occurred between two carp species estimated to have occurred 12 MYA, with two distinguishable sub-genomes with expressed ISG ohnologues within both sub-genomes [5,15,90]. Sub-genome dominance in common carp is unclear, with inferences ranging from the presence of sub-genome biased gene expression (P [91]) to no sub-genome dominance [5].

The key aim of this study was to examine the transcriptional response of the two common carp cell lines, CCB and KF1, in response to stimulation with the viral dsRNA mimic poly I: C. Both cell lines showed a characteristic interferon response suggesting that antiviral immune receptor and signalling pathways are functional in both cell lines. CCB consistently showed a more pronounced immune gene response compared to KF1.

## Materials and Methods

### Cell culture

Common Carp Brain (CCB) and Koi Fin-1 (KF1) cell lines were supplied by the Centre for Environment, Fisheries and Aquaculture Science (CEFAS) [33,67]. Cells were grown at 20°C in 75 cm^2^ flasks and trypsinised prior to passage every 14 days. Cells were split in a ratio of 1:2 and maintained in Eagle’s Minimum Essential Media, supplemented with 25mM HEPES, 2.2 g/L sodium bicarbonate, 2mM L-glutamine, 1% penicillin-streptomycin, 1% Non-Essential Amino Acids (Sigma-Aldrich), and 5% Foetal Bovine Serum.

### Stimulation of CCB and KF1 cells with the viral mimic poly I:C

For both cell lines, 2.5 × 10^6^ cells were plated into 12-well plates to a final volume of 2ml per well and incubated for 24h at 20°C to ensure full cell adhesion and recovery prior to immune stimulation. In line with previous studies examining the poly I:C mediated antiviral responses in fish primary cell and cell line cultures, CCB and KF1 cells were stimulated with 100 µg/ml of extracellular poly I:C for a 24h period [1,69]. Poly I:C (Sigma) was dissolved in water then diluted into cell culture media to a concentration of 100 µg/ml. For cell line stimulation, media was removed from each well and 2 mls of media containing poly I:C media was applied, followed by a further 24h incubation. Concurrently, time-matched non stimulated controls were set up and maintained as described or stimulated cells. Conditions and timepoints were replicated with n = 3.

### RNA Extraction

Cell media was removed from all wells and the adhered cells washed using 1 ml of cold 1xHBSS and overlaid with 1 ml of cold TRIzol (Invitrogen). Cell lysate from each well was transferred to 1.5 ml Eppendorf tubes and maintained at -80°C prior to RNA extraction. RNA was purified according to the manufacturers protocol. Precipitated RNA was washed twice in 500 µL 80% ethanol, dissolved in 50 µL RNAse free water, maintained on ice for 30 min, and incubated at 65°C for 5 min to completely dissolve the RNA.

RNA quantification was carried out on a NanoDrop™ One/OneC Microvolume UV-Vis Spectrophotometer (Thermo Fisher). RNA integrity was determined by Novogene Ltd. prior to sequencing using an Agilent 2100 Bioanalyser (Agilent Technologies), according to the manufacturer's instructions.

### RNA-Sequencing

Preparation of poly(A)+-enriched RNA transcripts from each sample and subsequent sequencing was performed by Novogene Ltd using a 150bp paired-end PE150 strategy. Total RNA (200ng) in 10 µL was used to create each library. Sequencing was carried out using a NovaSeq X Plus sequencing system at a depth of 30M reads per sample.

### Differential Gene Expression Analysis

Raw RNA-seq data was processed using NextFlow (v23.10.0) [18] and the NFcore RNAseq pipeline (v3.13.2) [21]. The STAR aligner option within NFcore was used to perform local alignments and quantification undertaken using the RSEM software package [52]. The RNA-seq data was stranded and adapter and quality trimming carried out using Trim Galore [47]. RNAseq reads were mapped to the common carp genome assembly, Cypcar_WagV4.0 (GCA_905221575.1) available via Ensembl [5]. The subread featureCounts program was used to generate raw counts using; the -p option to specify paired-end reads, the -B option to specify that fragments should only be summarised if both ends were aligned, and the -C option to prevent chimeric fragment inclusion [53]. The code utilised can be found in Appendix 1. Quality control of raw counts and mapped reads was performed using the MultiQC package in order to graphically visualize outputs [20].

Gene counts were processed using the DESeq2 package for R Studio to identify differentially expressed genes (DEGs). The samples, following mapping against 54,973 genes in the CypCar_WagV4.0 genome, were prefiltered to remove genes with a total row count < 10. Gene selection for DEG analysis was performed based on the following criteria: Benjamini-Hochberg adjusted p-value < 0.05, log_2_ fold change > 1 to represent upregulated genes, log_2_ fold change < -1 to represent downregulated genes. Gene annotations were generated through matching Ensembl gene IDs with corresponding HUGO Genome Nomenclature Committee (HGNC) IDs for protein-coding genes obtained from the human genome GRCh38.p14 (GCA_000001405.29) [59]. This was followed by human to carp gene annotations via Ensembl BioMart [45].

Overrepresentation analysis for functional biological process-associated groups was undertaken via gene ontology analysis.

Gene Ontology enrichment analysis was carried out using DEGs annotated with an HGNC ID, as listed above. Enrichment for biological process (BP) associated terms was performed using DAVID [34,76], utilising a standard background set of human genes. Up and down regulated gene lists were compiled and used to assess for enrichment of biological processes. Interpretation of BP GO terms was performed by using the Benjamini-Hochberg Procedure-controlled p-value (p<0.05) in order to assess significance. Visualisation of GO data was performed using the R package ggplot2 [87], with data manipulation performed using the tidyverse package [88].

### KEGG pathway analysis

Annotated DEG IDs for upregulated (padj < 0.05, L2FC > 1) and downregulated (padj < 0.5, L2FC < -1) transcripts were used to perform KEGG pathway analysis within DAVID [34,38,39,76].

## Results

### Overview of sequencing analysis

Sequencing returned an average of 36M reads for CCB cells and 34M reads for KF1 cells. Following sequence trimming, 31 to 41M reads and 31 to 48M reads were returned for CCB and KF1 libraries respectively, with 89.9% of reads for CCB, and 83.5% reads for KF1 mapping to the genome. Overall, 32.2-39.9% of the alignments were assigned to reads in all samples (Supp. Table 1). Raw data was submitted to the European Bioinformatics Institute under accession E-MTAB-15778.

**Figure 1:**
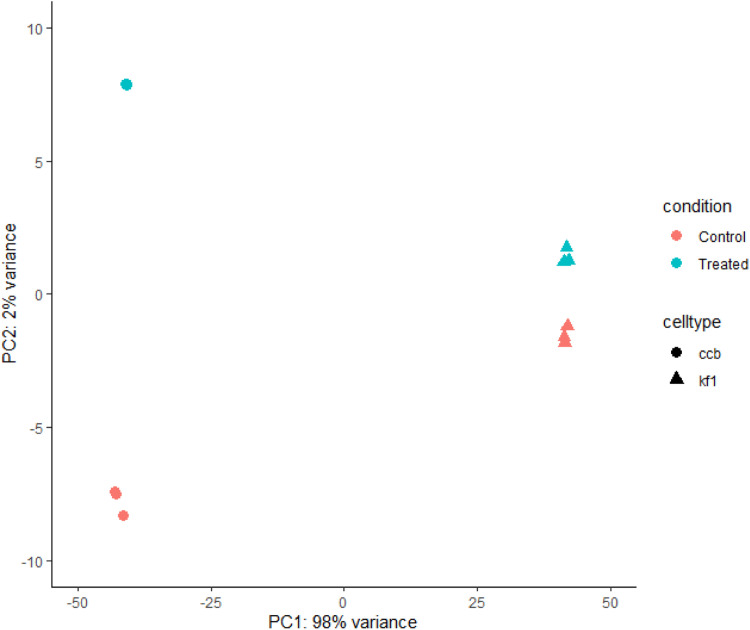
A PCA plot simultaneously showing poly I:C stimulated and control CCB and KF1 cells. n=3 for each group.

Following sequence filtering, 30,644 and 27,203 transcripts were retained for CCB and KF1 for differential gene expression analysis respectively Fig. 1.

### Analysis of Differentially Expressed Transcripts

To assess the magnitude of global differences between the CCB and KF1 cell lines and the responses to poly I:C stimulation, PCA plots were generated. PCA analysis clearly demonstrated a large (98% PC1 variance) difference between the cell lines, with a smaller (2% PC2 variance) difference observed for both cell lines due to poly I:C stimulation.

**Figure 2:**
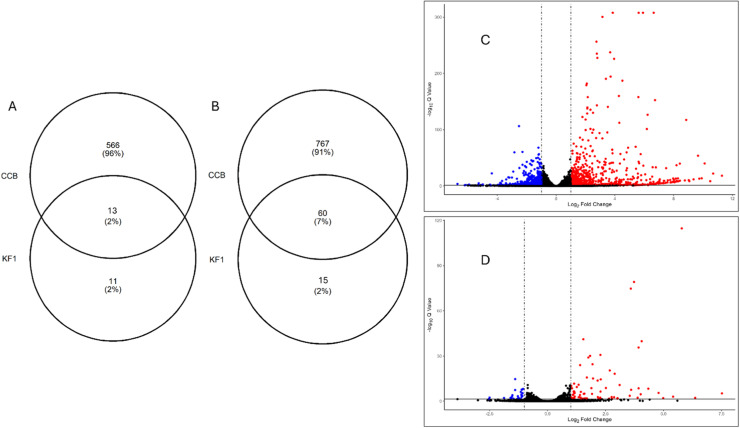
(A,B) Venn diagrams of downregulated (A) and upregulated (B) transcripts in CCB and KF1 after 24 hours of poly I:C stimulation. Bracketed numbers denote the percentage of transcripts in each section from the total pooled quantity of transcripts. (C, D) Volcano plots of differentially expressed transcripts in CCB (C) and KF1 (D) following 24h of poly I:C stimulation. Cutoffs are established at 1 and -1 log2 fold changes. P-values are adjusted using the Benjamini-Hochberg procedure. Red points represent a L2FC > 1, blue points represent L2FC < -1.

Following 24h of poly I:C stimulation, a total of 827 transcripts were found upregulated in CCB, and 75 in KF1 with 60 transcripts upregulated in both cell lines. Similarly, 579 and 24 transcripts were downregulated following poly I:C stimulation in CCB and KF1 cells with 13 transcripts downregulated in both cell lines (Figure 2).

### Determination of HGNC identifiers for annotated transcripts

All differentially expressed transcripts were annotated and assigned to HGNC identifiers enabling downstream functional enrichment of transcript sets. For the CCB cell line, 499 upregulated and 429 downregulated transcripts were assigned a corresponding HGNC ID. Whilst for KF1, only 48 upregulated and 15 downregulated transcripts were assigned an HGNC ID. For the purposes of multiple enrichment, redundant HGNC identifiers were removed, leaving 404 upregulated and 388 downregulated CCB transcripts and 42 upregulated and 12 downregulated for KF1 (Table 1).

**Table 1:** Summary of differentially expressed transcripts in CCB and KF1 cells following poly I:C stimulation and corresponding number of HGNC IDs in relation to human orthologs. Unique IDs indicate the total number of transcripts accounting for paralogous duplicated HGNC IDs, which are accounted as a single instance of a transcript.

Cell type	No. upregulated transcripts	No. HGNC IDs	No. unique HGNC IDs	Transcript copies	No. downregulated transcripts	No. HGNC IDs	No. unique HGNC IDs	Transcript copies
CCB	827	499	404	95	579	429	388	41
KF1	75	48	42	8	24	15	12	6

**Figure 3:**
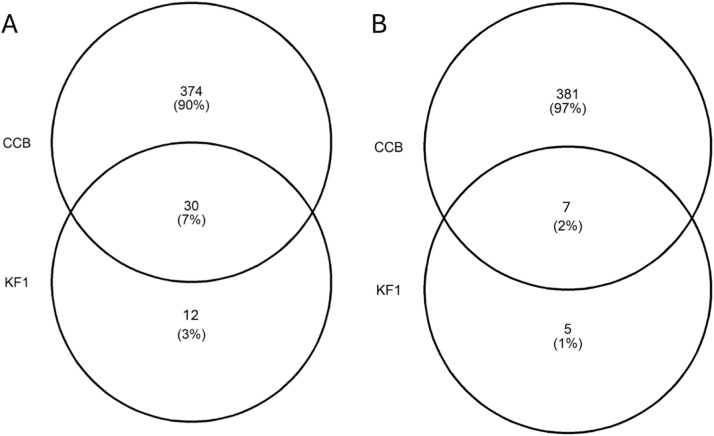
Upregulated (A) and downregulated (B) transcripts in poly I:C stimulated CCB and KF1 with annotated HGNC identifiers derived from Ensembl. The number of transcript IDs is indicated with numbers in parentheses denoting the proportion (%) of cell line -specific or common transcripts relative to the total number of transcripts.

Analysis of shared transcripts with unique HGNC identifiers between CCB and KF1 cells uncovered 374 unique upregulated HGNC IDs in CCB and 12 unique in KF1, with 30 common to both cell lines (Figure 3).

Transcripts upregulated in both stimulated cell lines are detailed in Table 2. The most upregulated transcript in CCB (ENSCCRG00000072503) represents an unannotated 168 amino acid open reading frame encoded by 5 exons. For most common transcripts (58/60), fold change was higher in CCB than in KF1 with most related to anti-viral immune responses. Among the common transcripts were some of the most highly up-regulated in both cell lines, such as ENSCCRG00000077198 (Interferon-Stimulated Gene 15/ISG15), ENSCCRG00000058239 (Interferon Induced Protein with Tetratricopeptide Repeats 1B/IFIT1B), and ENSCCRG00000067473 (Zinc Finger NFX1-Type Containing 1/ZNFX1) (Table 2). Only two upregulated common transcripts were more highly expressed in KF1 than CCB (ENSCCRG00000075971; Nuclear Factor, Erythroid 2/NFE2) and ENSCCRG00000055170; Collagen Type XV Alpha 1 Chain/COL15A1).

**Table 2:** Upregulated transcripts common to CCB and KF1 cell lines following poly I:C stimulation. ^a^Log^2^ Fold Change. A L2FC cutoff of >1 was applied. Green and red indicate > and < L2FC respectively in comparing the CCB response to poly I:C relative to KF1.

Many of the differentially expressed transcripts are members of gene families possessing multiple paralogues, including ISG15, IFIT1B, and TANK, that differ between CCB and KF1 cells in terms of the magnitude of differential expression. Of particular note were the paralogues of ISG15 in being highly variable with ENSCCRG00000077198 (10.49 L2FC in CCB and 4.35 L2FC in KF1) more highly expressed than ENSCCRG00000048369 (2.31 L2FC in CCB and 1.91 L2FC in KF1).

The 13 transcripts defined as downregulated in both CCB and KF1 are detailed in Table 3. This group includes; two paralogues of *col10a1* (ENSCCRG00000082308 and ENSCCRG00000079131, both encoding the alpha chain of type X collagen in humans) and two paralogues of the tetraspanin, *CD81b* (ENSCCRG00000044686 and ENSCCRG00000026566). Following transcript annotation, *Col10a1* and *CD81b* were not found to be associated with antiviral immune responses. However, it should be noted that *col10a1* expression is associated with human tumour cell activity and so may be linked to the immortalised status of these cell lines [75].

**Table 3:** Downregulated transcripts common to CCB and KF1 cell lines following poly I:C stimulation. ^a^Log^2^ Fold Change. A L2FC cutoff of >1 was applied. Green and red indicate > and < L2FC respectively in comparing the CCB response to poly I:C relative to KF1.

The top 20 most up- and down-regulated transcripts with a corresponding HGNC ID were examined. Further analysis utilised the full set of up- or down-regulated transcripts if not explicitly mentioned. In CCB, the most upregulated transcript was heat shock protein family B (small) member 7 (HSPB7) exhibiting a 10.69 L2FC fold change. Many immune response genes were found to be highly upregulated, including interferon-stimulated gene 15 (ISG15) at 10.49 L2FC and Interferon Induced Protein with Tetratricopeptide Repeats 1B (IFIT1B) at 9.66 L2FC. The most downregulated transcript was EGF like repeats and discoidin domains 3 (EDIL3) at -6.71 L2FC. In KF1, the most upregulated transcript was lymphotoxin alpha (LTA) at 6.37 L2FC, with other highly upregulated transcripts being SAA1 at 4.98 L2FC and ISG15 at 4.35 L2FC. The most downregulated transcript was Atonal bHLH transcription factor 8 (ATOH8) at -1.55 L2FC (Supp. Tables 4-7).

### Enriched functional ontology in common transcripts

**Figure 4:**
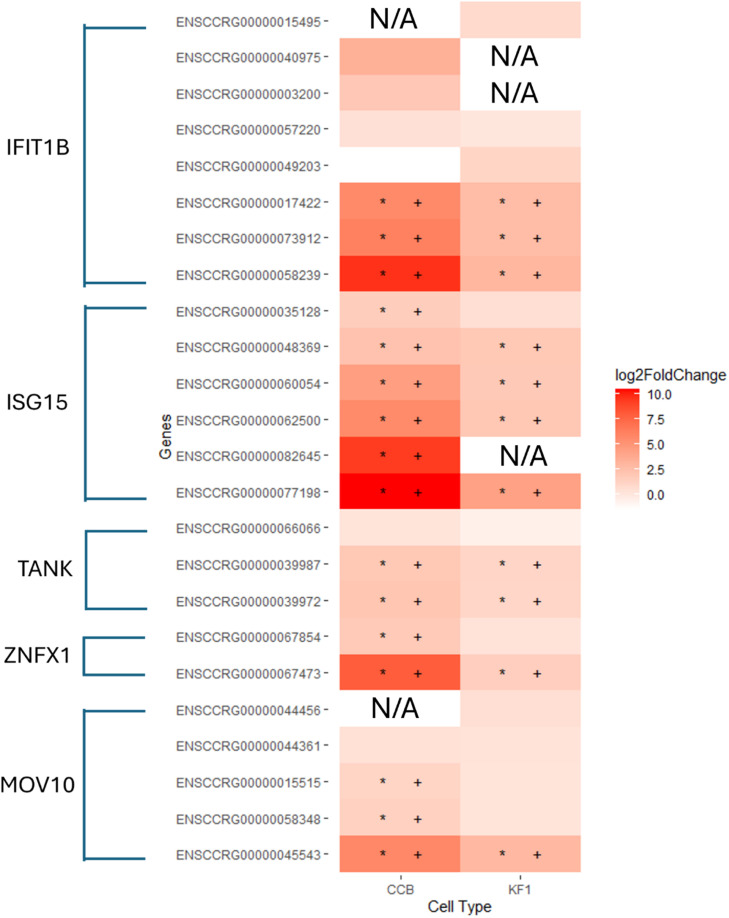
Heatmap of Ensembl transcript IDs associated with the term " defence response to virus” in upregulated transcripts common to CCB and KF1 cell lines. HGNC identifiers associated with each paralogue are appended to transcript IDs. * = padj < 0.05. + = L2FC > 1. N/A indicates removed transcripts due to very low representation in CCB and KF1 libraries (ie. <10 total counts prior to sequence filtering). IFIT1B: interferon induced protein with tetratricopeptide repeats 1B. ISG15: ISG15 ubiquitin like modifier. TANK: TRAF family member associated NFKB activator. ZNFX1: zinc finger NFX1-type containing 1. MOV10: Mov10 RNA helicase.

**Figure 5:**
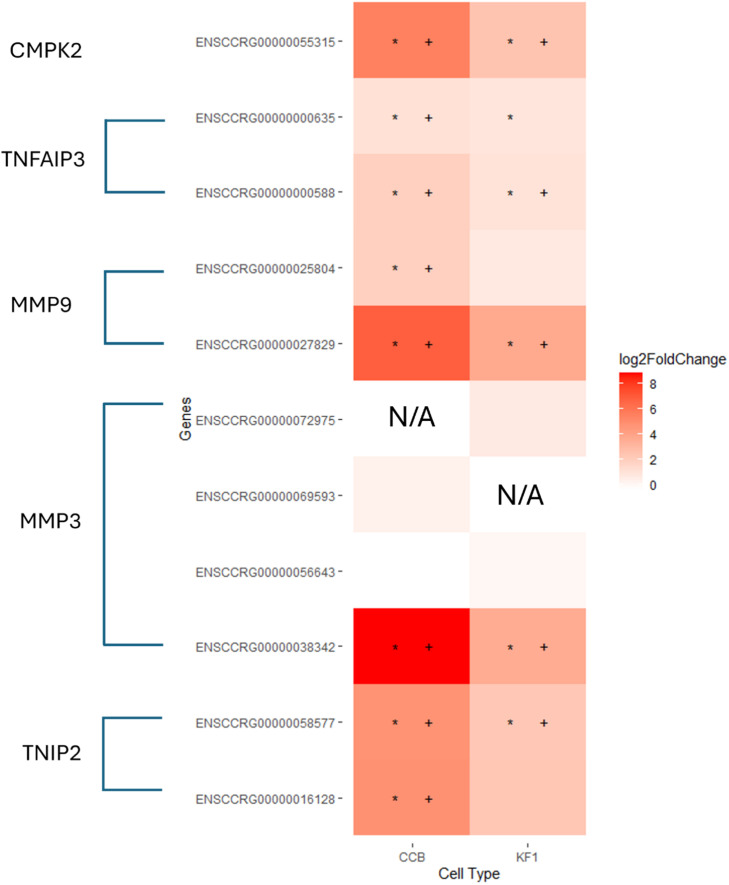
Heatmap of Ensembl transcript IDs associated with the term "cellular response to lipopolysaccharide" in upregulated transcripts common to CCB and KF1 cell lines. HGNC identifiers associated with each paralogue are appended to transcript IDs. * = padj < 0.05. + = L2FC > 1. N/A indicates removed transcripts due to very low representation in CCB and KF1 libraries (ie. <10 total counts prior to sequence filtering).

To further examine the functional groups of differentially expressed transcripts in CCB and KF1 in response to poly I:C, Gene Ontology analysis was performed. For transcripts found in both cell lines, two biological processes were identified, namely *“defence response to virus”* (GO:0051607) and *“cellular response to lipopolysaccharide”* (GO:0071222) (Figures 4 & 5).

### Overrepresentation analysis in CCB and KF1 by gene ontology

Following enrichment analysis, 23 Biological Process GO (BP GO) terms derived from upregulated transcripts were identified in CCB cells, and 4 BP GO terms from stimulated KF1 cells. No significant GO BP terms were identified from downregulated transcripts in either CCB or KF1.

**Figure 6:**
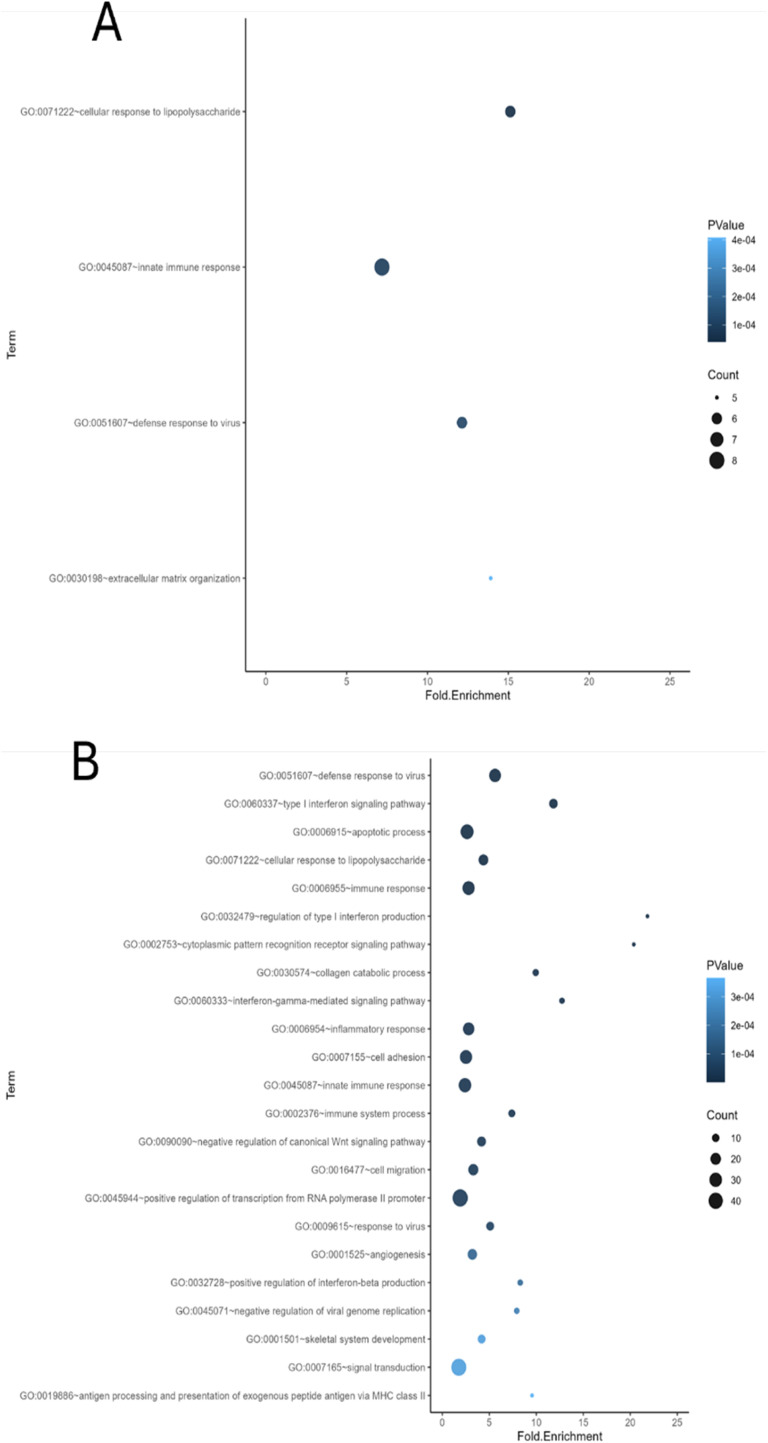
A bubble plot display of significant gene ontology terms calculated from HGNC-annotated upregulated transcripts in poly I:C stimulated KF1 (A) and CCB (B) cells ordered by ascending significance.

For KF1 cells the most enriched functions were *“defence response to virus”* (GO:0051607), *“cellular response to lipopolysaccharide”* (GO:0071222), *“innate immune response”* (GO:0045087), and *“extracellular matrix organization”* (GO:0030198) (Figure 6A).

In stimulated CCB cells, the most significantly enriched term was *“defence response to virus”* (GO:0051607), followed by *“type I interferon signalling pathway”* (GO:0060337) and *“apoptotic process”* (GO:0006915). Other key immune terms enriched included *“immune response”* (GO:0006955), *“regulation of type I interferon production”* (GO:0032479), *“interferon-gamma-mediated signalling pathway”* (GO:0060333), and *“innate immune response”* (GO:0045087) (Figure 6B).

### Defence response to virus as a key Gene Ontology term

To examine potential differences in paralogue expression and cell line-specific changes, transcripts within the functional group common to both cell lines (*“defence response to virus”* GO:0051607) were chosen. 26 transcripts were assigned to this GO term in CCB. Gene ontology was determined using the DAVID program with HGNC identifiers as inputs. Thus, each identifier was assigned to individual gene paralogues yielding 49 significantly upregulated transcripts due to WGD events. In KF1, we found a total count of 6 upregulated transcripts associated with *“defence response to virus”* (GO:0051607), expanding to a total of 12 transcripts following identification of gene paralogues.

Several transcripts supported by multiple paralogues within this group represent well characterised ISGs, including DHX58, with four paralogues, ISG15 with seven paralogues, and IRF3 and IRF1 with two paralogues each. For CCB and KF1 ISG15 was found to be represented by four differentially expressed paralogues. Furthermore, as in CCB, three paralogues were designated as IFIT1B in KF1 cells. Only a single paralogue of MOV10 was upregulated in KF1, compared to three in CCB. All MOV10 paralogues were found to be upregulated following poly I:C stimulation.

### Toll-like receptor 3 expression in CCB & KF1

To clarify the differential antiviral response in CCB and KF1, we examined the expression of the primary dsRNA receptor TLR3 in response to poly I:C (Table 4). HGNC annotation of the Ensembl common carp genome did not annotate any TLR3 paralogues, although two copies of TLR3 were already found to be annotated within the genome (ENSCCRG00000078962 & ENSCCRG00000059536) with no further paralogues identified through BLAST analysis. Neither copy was significantly differentially expressed within CCB or KF1, but were differentially expressed between CCB and KF1. The base mean of both TLR3 paralogues was higher in CCB than KF1, with ENSCCRG00000078962 showing higher counts than ENSCCRG00000059536.

**Table 4:** Expression of TLR3 in CCB and KF1 following poly I:C stimulation. Base mean is an average of the normalised count values divided by size factors. L2FC = Log2 Fold Change.

Ensembl Transcript ID	Gene ID	Base Mean cpm (CCB)	L2FC (CCB)	Adjusted p-value (CCB)	Base Mean cpm (KF1)	L2FC (KF1)	Adjusted p-value (KF1)	Sub-genome
ENSCCRG00000059536	*tlr3*	380.19	0.71	0.00045	16.41	0.59	0.95897	A
ENSCCRG00000078962	*tlr3*	1226.28	-0.19	0.51901	314.15	0.02	0.99699	B

### KEGG analysis in CCB and KF1

**Figure 7:**
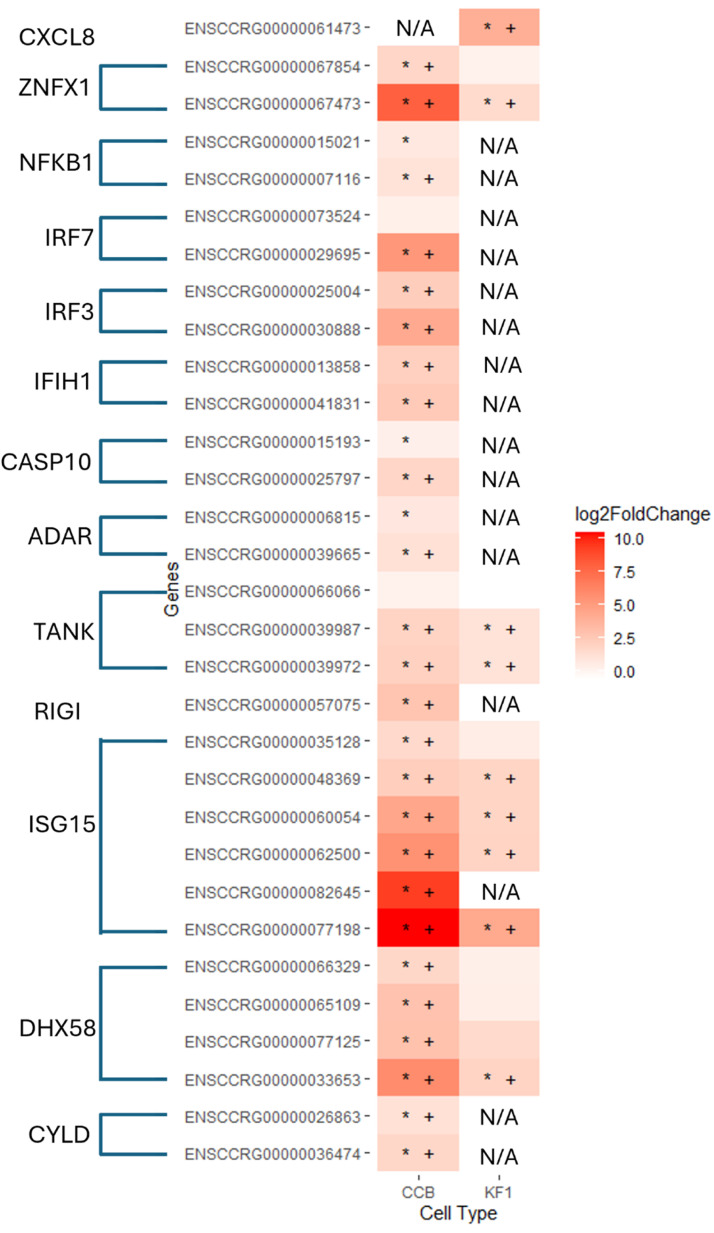
Expressed transcripts involved in the KEGG pathway “RIG-I-like receptor signalling pathway” in CCB and KF1 cells. * = padj < 0.05, + = L2FC > 1, N/A = HGNC IDs not found due to transcripts not being differentially expressed.

For CCB cells, 18 KEGG pathways were significantly enriched (Supp. Table 9), including “*RIG-1-like receptor signalling pathway*” with 12 out of the 72 genes involved in the RIG-1 signalling pathway represented in the upregulated transcript set. This included the core set of receptors involved in viral dsRNA recognition (RIG-I, MDA5, and DHX58) and the key antiviral transcription factors IRF3 and IRF7 (Supp. Figure 1). For KF1, only “*RIG-1 like receptor signalling pathway*” was significantly enriched being represented by 5 transcripts. The representative transcripts for RIG1 pathway expression are presented in Figure 7.

## Discussion

The key aim of this study was to investigate the innate antiviral immune capacity of the CCB and KF1 cell lines at the transcriptome level in response to the commonly used viral dsRNA mimetic, (poly I:C). Overall, we uncovered major differences between the magnitude and intensity of the antiviral immune response in CCB compared with KF1, highlighting the importance of ongoing cell line characterisation and assessment in immunological studies. CCB and KF1 cells are both used for virus research. CCB has been used in assessing the inhibitory capability of lactobacillus metabolites on CyHV-3 [13], in assessing edited CyHV-3 replication during infection [56], and in determining that CyHV-3 altered autophagy during the cycle of infection [61]. KF1 has been used in assessing the cytotoxicity of the antiviral compounds acyclovir and cidofovir [70] and the antiviral efficacy of thyme oil against CyHV-3 [82]. Furthermore, current usage of these cell lines for CyHV-3 culture and diagnostics highlights their importance for viral characterization and therapeutic intervention, as recommended by the World Organisation for Animal Health [89].

Representation of interferon-inducible antiviral genes in differentially expressed transcripts common to both poly I:C stimulated cell lines is strongly indicative that CCB and KF1 are able to mount an interferon driven antiviral response. Our results agree with the activation of other immune response associated terms in the top 20 GO terms for CCB, such as “*type I interferon signaling pathway”* (GO:0060337), where we observed upregulation of interferon alpha and beta receptor subunit 1 (IFNAR1). This is an important receptor of type-I interferons with its loss of function associated with impeded interferon signalling in humans [95]. Furthermore, the term “*cytoplasmic pattern recognition receptor signaling pathway*” (GO:0002753) was upregulated, including transcripts, such as RIGI, DHX58, IRF3, and IRF7. Together with representation of the term “*innate immune response*” (GO:0045087), this confirms that poly I:C induced an antiviral immune state characterized by a type-1 interferon response in CCB, as reported previously [1].

### Cell lines vary in antiviral response

Our results are in line with previous observations that the susceptibility and sensitivity of cell lines to stimulation by viral pathogens can be highly variable. Hence, appropriate molecular characterisation is key to choosing the optimal cell line accounting for host and organ specificity. Iridovirus stimulation in three Japanese seabass (*Lateolabrax japonicu*) brain-derived cell lines (LMB-S, LMB-M, and LMB-L) exhibit significant variation in JAK1, STAT1, STAT2, and IRF9 expression. Phenotypically, these cell lines vary in terms of cell adherence and cell heterogeneity, that likely accounts for observed differences in viral replication capacity [57]. Cell lines established from goldfish and silver crucian carp (*Carassius carassius*) spleen, kidney, and fin tissue were not able to permit the proliferation of CyHV-2. Whilst, CrCB and GFB cell lines, established from the brain tissue of these fish species, were permissive to replication [91].

Previous carp fin-derived cell lines have been successfully used in viral propagation, such as the Koi Fin-101 (KF-101) cell line which demonstrated typical CPE effects when exposed to CyHV-3 [55]. Similarly, a caudal fin cell line from koi (KCF-1) also supports CyHV-3 replication [19], further demonstrating the importance of cell line selection and establishment for viral research.

Viral tropism is an overarching field that describes the specificity of viruses for different hosts, tissues, and cell types, and is a key factor in considering suitable cell culture systems in virology [63]. Viruses depend on the presence of cell-surface receptors to gain entry, such as in Human Immunodeficiency Virus 1 (HIV-1), which requires the presence of CD4 and chemokine receptors, CXCR4 and CCR5 to bind and enter cells [3]. Another key factor can be whether a cell line can mount an effective type-I interferon-driven antiviral immune response to viral infection [93]. Determining the factors required for cellular entry can be complicated by viruses with specific host tropisms, but permissive replication within cells derived from a range of species. This is particularly of note with relation to CyHV-3, which induces cytopathic effects in cell lines derived from a range of species, including silver carp and goldfish [[16], [92]].

The cell line CCApin originates from common carp fin tissue and showed a differing pattern in interferon response when exposed to a viral challenge in comparison to CCB and cell lines sourced from common carp gill and heart [26]. Similarly, a notable example of a cell line lacking antiviral responsiveness to poly I:C stimulation is the Chinook Salmon Embryo (CHSE-214) cell line, which lacks the ability to respond to non-transfected poly I:C stimulation [36]. This was suggested to be due to a loss of scavenger receptors involved in the internalisation and binding of extracellular dsRNA [65], but may also reflect low abundance or absence of TLR3 in this cell line. In contrast, the Atlantic salmon (*Salmo salar*) fin tissue cell line, ASF is susceptible to Nervous necrosis virus (NNV) further emphasizing the diversity of available cell lines for fish viral research [37]. Overall, these results underline the importance of cell line characterization in the context of viral research. CCB and KF1 are well established as relevant and susceptible to CyHV-3 viral infection [33,67].

### Poly I:C mediated transcriptional modulation of interferon response genes in CCB and KF1 cell lines

In mammals, detection of viral dsRNA occurs via MDA5 and TLR3 driven pathways. Upon binding to viral dsRNA, conformational changes lead to exposure of the Caspase recruitment domains (CARDs), enabling interaction with the mitochondrial antiviral signalling (MAVS) protein, IPS-1 [42]. MAVS activation, in turn, activates NF-κB, IRF3, and IRF7 pathways to establish an antiviral state capable of inhibition of viral replication [23,74]. This is achieved through the activation of type-I IFN transcription and downstream induction of ISGs. Similarly, dsRNA binding to TLR3 triggers activation of these mechanisms via the Toll / IL-1 receptor domain containing adapter inducing interferon β (TRIF) pathway [7].

In this study, the endosomally located receptor TLR3 was examined due to its importance in the detection of extracellular poly I:C [11]. Expression of two Ensembl-annotated TLR3 paralogues in CCB and KF1 differed and was more abundant in CCB compared to KF1. However, we did not find TLR3 mRNA expression to be upregulated significantly in either CCB or KF1. This is particularly notable in the context of stimulation via poly I:C, as poly I:C is known to strongly induce TLR3 activation in mammals [54] and fish [22]. Despite the lack of marked poly I:C mediated expression of both paralogues, paralogue ENSCCRG00000078962 from sub-genome B, was more abundant than ENSCCRG00000059536 on sub genome A in both cell lines. The higher expression of TLR3 in CCB may explain the increased responsiveness to poly I:C stimulation compared to KF1 by facilitating signalling through the type-I IFN pathway. A similar response to poly I:C is observed in human neuroblastoma cells where cells with high TLR3 levels express enhanced activation and expression of EIF2AK2 in comparisons to cells with low expression of TLR3 [8].

The receptors IFIH1 and RIGI, among the core transcripts representing the RIG-I pathway in our datasets, were upregulated in CCB but not in KF1. The RIG-I signalling pathway is crucial in inducing the production of type-I IFN and establishing an antiviral state following detection of viral RNA [41]. Higher basal and poly I:C mediated expression of RIGI and IFIH1 in CCB aligns with previous observations for human RIGI. Here, the late-phase stage of the RIGI induction process leads to the accumulation of RIGI within the cytosol, although the ultimate function of accumulated RIGI may not relate to antiviral signalling [79]. This has not currently been explored in IFIH1. As the initial immune response is driven by the constitutively expressed RIGI and MDA5 proteins, the absence of upregulated expression in KF1 cells in this study does not preclude their role in the antiviral response.

Several transcripts found in the core response are involved in the Nuclear Factor‐κB (NF-κB) pathway, that is critical to early pathogen-mediated immune responses. This includes regulators and promoters such as TANK [85], TNFAIP3 [86], and REL [73]. TLR3/MDA-5 mediated induction of the NF-kB pathway is well established in mammalian models, where deficient TLR3 ablation leads to a loss of poly I:C responsiveness in fibroblastic cells, with impaired NF-kB and IRF3 activation [96]. Such observations may, to some extent, account for the low poly I:C responsiveness of KF1 cells due to a dampening of the NF-κB pathway by sub-optimal expression of TLR3.

Tumour necrosis factor receptor-associated factor (TRAF) family member-associated NF-κB activator (TANK) is important to NF-κB pathway activation and antiviral immune responses [31]. TANK is a key regulator of TLR-induced antiviral pathways inhibiting NF-κB activity mediated by TNF Receptor Associated Factor 6 (TRAF6) [9,35]. Upregulation of TANK in CCB and KF1 in this study is consistent with previous findings in poly I:C stimulated European sea bass (*Dicentrarchus labrax*) [83] and large yellow croaker (*Pseudosciaena crocea*) [66].

Amongst the transcripts upregulated in both CCB and KF1 was ISG15, an interferon-stimulated ubiquitin-like protein which has a major role in the ISGylation of target proteins, [40]. Through ISGylation of STAT1, ISG15 promotes IFN responses by enhancing activation of downstream components via phosphorylation [28]. Furthermore, ISG15 has a role in negative regulation of the type-1 IFN response through ISGylation of the RIG-1 protein [44]. ISG15 attenuation via type-I IFN receptor knock out in mice highlights the importance of IFN to the ISG15 response [49].

MOV10 expression was activated in both CCB and KF1. Mov10 is a multifunctional protein modulated by IRF3 targeting RNA viruses, potentially via RNA binding in forming complexes with IFIT proteins [12]. Overexpression of MOV10 in the Japanese sea bass cell line, LJF inhibited VHSV replication through the perciform-specific interferon, IFNh [60]. MOV10 has also been identified as a key element of a novel innate RNAi response in ISAV infected Atlantic salmon [80]. Upregulation of MOV10 in CCB and KF1 cells is consistent with known antiviral immune responses to poly I:C [84]. A further commonly induced ISG in CCB and KF1 was IFIT1B that suppresses viral replication via binding to 2’O unmethylated capped viral RNA [32] and recognition of unmethylated mRNA [14].

Upregulation of ISG15, MOV10, and IFIT1B strongly support the notion that both cell lines undergo antiviral activation following poly I:C stimulation, although the lower response in KF1 could reflect differences in the tissue source of each cell line. Central nervous system tissues such as the brain can show unique responses [58], which may help explain differences between the brain-derived CCB and the fin-derived KF1. Viral infection with a nodavirus in sea bream (*Sparus auratus*) and European seabass induced an antiviral and proinflammatory response in brain tissues from these fish [68]. Furthermore, infectious hematopoietic necrosis virus (IHNV) infection in rainbow trout (*Oncorhynchus mykiss*) induced a strong antiviral response within brain tissue [78].

Although we did not find evidence for TNF-α upregulation in CCB, we do find the family member TNFAIP3 upregulated, which is involved in inflammasome signalling in mice [46]. The disparity between HGNC annotation and the Cypcar_WagV4.0 annotation id is particularly evident in the case of IFIT1B, wherein each instance of a gene here assigned to IFIT1B by HGNC annotation is assigned to a different gene within the ifit family within carp. It should also be noted that the upregulation of LTA here may instead represent the expression of isoforms of TNF-α, as LTA is not present in many fish, and should highlight the importance of improved annotation and functional studies.

Carp express 4 STAT1 paralogues, two copies of *stat1a* and *stat1b*, of which both copies of *stat1b* and one copy of *stat1a* were upregulated in CCB. This correlates with the occurrence of multiple upregulated ISGs seen in the “*defence response to virus*” term, such as ISG20 and EIF2AK2 both having potent antiviral properties ([17]; Chaumont et al., 2021) and IRF1, a core regulator of the interferon response [27]. The presence and marked upregulation of these ISGs indicates a sustained antiviral response in CCB, although their much lower expression in KF1 suggests a weaker antiviral response. Importantly, neither *stat1a* nor *stat1b* paralogues were upregulated in KF1 cells despite observing upregulation of multiple components of the RIG-I pathway.

### Paralogous gene induction in CCB and KF1, with specific reference to sub-genomes

When differentially expressed genes were examined for association with sub genomes A and B, we did not find any global trends for either CCB and KF1 cell lines. This is in agreement with Blasweiler *et al.*, [5], although Chen *et al.*, [6], concluded that sub genome specific differential expression was due to thermal stress responses. Current thinking suggests that the mechanism of heterosis results in hybrid offspring possessing multiple sub genomes. Thus, fish species, such as common carp, would favour high expression of dominant alleles where deleterious alleles from one parent are suppressed by dominant alleles from the other [4]. This would imply that the hybridised common carp may exhibit dominance of one sub genome over the other.

## Conclusions

Our results show that two commonly used carp cell lines CCB and KF1 both respond to the dsRNA mimetic, poly I:C. The magnitude of antiviral responses were more highly represented in CCB libraries with associated transcripts being expressed to a greater extent than in KF1 cells. Additionally, we examined the expression of paralogues derived from the carp-specific whole genome duplication, termed sub-genome A and sub-genome B, although there was no clear evidence supporting sub genome specific responses to poly I:C stimulation.

It is noted that the use of a single time point allows us to only observe a single static point of responses in the antiviral cascade; previous work with CCB cells in viral stimulation have found genes such as IRF3 and IRF7 to be upregulated within 6h of poly I:C stimulation, but declining by 12h, with *IFN-a* and *Vig1* upregulated as early as 2h post-stimulation [1]. The potential to observe early antiviral responses in KF1 is also lost by the use of the 24h timepoint, as immediate responses can vary between cell types, such as in the expression of IRF9 in common carp, whereby significant expression can vary widely from 3 hours in spleen to 48 hours in head kidney [97].

The differential responses observed between CCB and KF1 could be attributed to the differential expression of key transcriptional drivers of antiviral immunity or differences in the abundance and diversity of PRRs driving dsRNA mediated responses. It may also be attributed to differences in epigenomic factors, such a chromatin accessibility that could to some extent account for the transcriptional heterogeneity of CCB and KF1 cells. Importantly, however, the underlying molecular response to poly I:C in CCB and KF1 cells may also reflect differences in species and tissue origin, viral replication capacity, and cellular composition [63,72].

Overall, results of this study provide important insights into the underlying molecular mechanisms of the antiviral capacity of CCB and KF1 cell lines with relevance to ongoing immunological characterisation of other fish cell lines.

## Funding

This work was partly funded by a PhD studentship from the Centre for Environment, Fisheries, and Aquaculture Science [Defra contract FC1215].

## CRediT authorship contribution statement

**Laurie Smeaton:** Writing – review & editing, Writing – original draft, Methodology, Investigation, Formal analysis, Data curation. **Richard Paley:** Writing – review & editing, Supervision, Project administration, Investigation, Funding acquisition, Conceptualization. **Irene Cano Cejas:** Writing – review & editing, Supervision, Project administration, Methodology, Funding acquisition, Conceptualization. **Jason W. Holland:** Writing – review & editing, Supervision, Funding acquisition, Conceptualization. **Samuel A.M. Martin:** Writing – review & editing, Project administration, Funding acquisition, Conceptualization.

## Declaration of competing interest

The authors declare that they have no known competing financial interests or personal relationships that could have appeared to influence the work reported in this paper.

## Data Availability

Data will be made available on request.

## Appendix Supplementary materials

Image, application 1

## References

[1] Adamek M. (2012). Interferon type I responses to virus infections in carp cells: In vitro studies on Cyprinid herpesvirus 3 and Rhabdovirus carpio infections. Fish & Shellfish Immunology.

[2] Ashraf U. (2016). Spring viraemia of carp virus: recent advances. Journal of General Virology.

[3] Berger E.A., Murphy P.M., Farber J.M. (1999). CHEMOKINE RECEPTORS AS HIV-1 CORECEPTORS: Roles in Viral Entry, Tropism, and Disease. Annual Review of Immunology.

[4] Birchler J.A. (2010). Heterosis. The Plant Cell.

[5] Blasweiler A. (2023). Symmetric expression of ohnologs encoding conserved antiviral responses in tetraploid common carp suggest absence of subgenome dominance after whole genome duplication. Genomics.

[6] Chen L. (2024). Evolutionary divergence of subgenomes in common carp provides insights into speciation and allopolyploid success. Fundamental Research.

[7] Chen Y. (2021). Toll-like receptor 3 (TLR3) regulation mechanisms and roles in antiviral innate immune responses. Journal of Zhejiang University-SCIENCE B.

[8] Chuang J.-H. (2011). Differential toll-like receptor 3 (TLR3) expression and apoptotic response to TLR3 agonist in human neuroblastoma cells. Journal of Biomedical Science.

[9] Clark K. (2011). The TRAF-associated protein TANK facilitates cross-talk within the IκB kinase family during Toll-like receptor signaling. Proceedings of the National Academy of Sciences.

[10] Clouthier S. (2017). Diagnostic validation of three test methods for detection of cyprinid herpesvirus 3 (CyHV-3). Diseases of Aquatic Organisms.

[11] Collet B. (2026). Toll-like Receptor 3 is Required for Sensing Extracellular Double Stranded RNA in Salmonid Cells. Marine Biotechnology.

[12] Cuevas R.A. (2016). MOV10 Provides Antiviral Activity against RNA Viruses by Enhancing RIG-I–MAVS-Independent IFN Induction. The Journal of Immunology.

[13] Danova S. (2024). Lactobacilli-Derived Postmetabolites Are Broad-Spectrum Inhibitors of Herpes Viruses In Vitro. International Journal of Molecular Sciences.

[14] Daugherty M.D. (2016). Evolution-guided functional analyses reveal diverse antiviral specificities encoded by IFIT1 genes in mammals. eLife.

[15] David L. (2003). Recent Duplication of the Common Carp (Cyprinus carpio L.) Genome as Revealed by Analyses of Microsatellite Loci. Molecular Biology and Evolution.

[16] Davidovich M. (2007). Susceptibility of cyprinid cultured cells to cyprinid herpesvirus 3. Archives of Virology.

[17] Deymier S. (2022). ISG20: an enigmatic antiviral RNase targeting multiple viruses. FEBS open bio.

[18] Di Tommaso P. (2017). Nextflow enables reproducible computational workflows. Nature Biotechnology.

[19] Dong C. (2011). Characterization of a new cell line from caudal fin of koi, Cyprinus carpio koi, and first isolation of cyprinid herpesvirus 3 in China. Virus Research.

[20] Ewels P. (2016). MultiQC: summarize analysis results for multiple tools and samples in a single report. Bioinformatics.

[21] Ewels P.A. (2020). The nf-core framework for community-curated bioinformatics pipelines. Nature Biotechnology.

[22] Falco A. (2014). β-Glucan-supplemented diets increase poly(I:C)-induced gene expression of Mx, possibly via Tlr3-mediated recognition mechanism in common carp (Cyprinus carpio). Fish & Shellfish Immunology.

[23] Fang R. (2017). MAVS activates TBK1 and IKKε through TRAFs in NEMO dependent and independent manner. *PLOS Pathogens*. Edited by P. Feng.

[24] FAO (ed.) (2022) *Towards blue transformation*. Rome: FAO (The state of world fisheries and aquaculture, 2022). Available at: 10.4060/cc0461en.

[25] FAO (2023). Fishery and Aquaculture Statistics. Global production by production source 1950-2021 (FishStatJ)’. Rome: FAO Fisheries and Aquaculture Division.

[26] Felten M. (2022). The influence of viral infection on cell line characteristics: Lessons learned from working with new cell lines from common carp. Journal of Fish Diseases.

[27] Feng H. (2021). Interferon regulatory factor 1 (IRF1) and anti-pathogen innate immune responses. PLoS pathogens.

[28] Ganesan M. (2016). Acetaldehyde Disrupts Interferon Alpha Signaling in Hepatitis C Virus-Infected Liver Cells by Up-Regulating USP 18. Alcoholism: Clinical and Experimental Research.

[29] Glasauer S.M.K., Neuhauss S.C.F. (2014). Whole-genome duplication in teleost fishes and its evolutionary consequences. Molecular Genetics and Genomics.

[30] Gotesman M. (2013). CyHV-3: the third cyprinid herpesvirus. Diseases of Aquatic Organisms.

[31] Guo B., Cheng G. (2007). Modulation of the Interferon Antiviral Response by the TBK1/IKKi Adaptor Protein TANK. Journal of Biological Chemistry.

[32] Habjan M. (2013). Sequestration by IFIT1 Impairs Translation of 2′O-unmethylated Capped RNA. *PLoS Pathogens*. Edited by M.S. Diamond.

[33] Hedrick R.P. (2000). A Herpesvirus Associated with Mass Mortality of Juvenile and Adult Koi, a Strain of Common Carp. Journal of Aquatic Animal Health.

[34] Huang D.W., Sherman B.T., Lempicki R.A. (2009). Systematic and integrative analysis of large gene lists using DAVID bioinformatics resources. Nature Protocols.

[35] Huang L. (2015). Encephalomyocarditis Virus 3C Protease Relieves TRAF Family Member-associated NF-κB Activator (TANK) Inhibitory Effect on TRAF6-mediated NF-κB Signaling through Cleavage of TANK. The Journal of Biological Chemistry.

[36] Jensen I., Larsen R., Robertsen B. (2002). An antiviral state induced in Chinook salmon embryo cells (CHSE-214) by transfection with the double-stranded RNA poly I:C. Fish & Shellfish Immunology.

[37] Jia P. (2022). Establishment and Characterization of a Fin Cell Line Derived from the Atlantic Salmon Salmo salar and Its Application to Fish Virology Study. Journal of Ocean University of China.

[38] Kanehisa M. (2019). Toward understanding the origin and evolution of cellular organisms. Protein Science.

[39] Kanehisa M. (2025). KEGG: biological systems database as a model of the real world. Nucleic Acids Research.

[40] Kang J.A., Kim Y.J., Jeon Y.J. (2022). The diverse repertoire of ISG15: more intricate than initially thought. Experimental & Molecular Medicine.

[41] Kato H. (2008). Length-dependent recognition of double-stranded ribonucleic acids by retinoic acid–inducible gene-I and melanoma differentiation–associated gene 5. The Journal of Experimental Medicine.

[42] Kawai T. (2005). IPS-1, an adaptor triggering RIG-I- and Mda5-mediated type I interferon induction. Nature Immunology.

[43] Kawai T., Akira S. (2009). The roles of TLRs, RLRs and NLRs in pathogen recognition. International Immunology.

[44] Kim M.-J. (2008). Negative Feedback Regulation of RIG-I-Mediated Antiviral Signaling by Interferon-Induced ISG15 Conjugation. Journal of Virology.

[45] Kinsella R.J. (2011). Ensembl BioMarts: a hub for data retrieval across taxonomic space. Database.

[46] Kool M. (2011). The Ubiquitin-Editing Protein A20 Prevents Dendritic Cell Activation, Recognition of Apoptotic Cells, and Systemic Autoimmunity. Immunity.

[47] Krueger F. (2023). FelixKrueger/TrimGalore: v0.6.10 - add default decompression path. Zenodo.

[48] Kuzmin E., Taylor J.S., Boone C. (2022). Retention of duplicated genes in evolution. Trends in genetics: TIG.

[49] Lenschow D.J. (2005). Identification of Interferon-Stimulated Gene 15 as an Antiviral Molecule during Sindbis Virus Infection In Vivo. Journal of Virology.

[50] Levraud J.-P. (2019). IFN-Stimulated Genes in Zebrafish and Humans Define an Ancient Arsenal of Antiviral Immunity. The Journal of Immunology.

[51] Lewisch E. (2015). Carp Edema Virus/Koi Sleepy Disease: An Emerging Disease in Central-East Europe. Transboundary and Emerging Diseases.

[52] Li B., Dewey C.N. (2011). RSEM: accurate transcript quantification from RNA-Seq data with or without a reference genome. BMC Bioinformatics.

[53] Liao Y., Smyth G.K., Shi W. (2014). featureCounts: an efficient general purpose program for assigning sequence reads to genomic features. Bioinformatics.

[54] Lim C.S. (2022). TLR3 forms a highly organized cluster when bound to a poly(I:C) RNA ligand. Nature Communications.

[55] Lin S.-L. (2013). Characterization of a novel cell line from the caudal fin of koi carp *Cyprinus carpio*. Journal of Fish Biology.

[56] Liu D. (2025). Cyprinid herpesvirus 3 replication is inhibited via decreased ORF3 promoter activity mediated by multiple cellular transcription factors. Aquaculture.

[57] Liu Z., Ma Y., Hao L. (2022). Characterization of three novel cell lines derived from the brain of spotted sea bass: Focusing on cell markers and susceptibility toward iridoviruses. Fish & Shellfish Immunology.

[58] Louveau A., Harris T.H., Kipnis J. (2015). Revisiting the Mechanisms of CNS Immune Privilege. Trends in Immunology.

[59] Love M.I., Huber W., Anders S. (2014). Moderated estimation of fold change and dispersion for RNA-seq data with DESeq2. Genome Biology.

[60] Lu X. (2023). Molecular characterization, transcriptional regulation of sea perch Moloney leukemia virus 10 and its antiviral function against VHSV. Fish & Shellfish Immunology.

[61] Luo W. (2023). Autophagy induced by Cyprinid herpesvirus 3 (CyHV-3) facilitated intracellular viral replication and extracellular viral yields in common carp brain cells. Fish & Shellfish Immunology.

[62] Machat R. (2021). Carp edema virus and immune response in carp (*Cyprinus carpio*): Current knowledge. Journal of Fish Diseases.

[63] McFadden G. (2009). Cytokine determinants of viral tropism. Nature Reviews Immunology.

[64] Michel B. (2010). Cyprinid Herpesvirus 3. Emerging Infectious Diseases.

[65] Monjo A.L., Poynter S.J., DeWitte-Orr S.J. (2017). CHSE-214: A model for studying extracellular dsRNA sensing in vitro. Fish & Shellfish Immunology.

[66] Mu Y. (2014). De Novo Characterization of the Spleen Transcriptome of the Large Yellow Croaker (Pseudosciaena crocea) and Analysis of the Immune Relevant Genes and Pathways Involved in the Antiviral Response. *PLoS ONE*. Edited by F.C.C. Leung.

[67] Neukirch M., Bottcher K., Bunnajirakul S. (1999). Isolation of a virus from koi with altered gills. Bulletin of the European Association of Fish Pathologists.

[68] Poisa-Beiro L. (2008). Nodavirus increases the expression of Mx and inflammatory cytokines in fish brain. Molecular Immunology.

[69] Porter D. (2022). Gut Associated Lymphoid Tissue (GALT) primary cells and stable cell lines as predictive models for intestinal health in rainbow trout (Oncorhynchus mykiss). Frontiers in Immunology.

[70] Quijano Cardé E.M. (2020). Pharmacokinetic and Efficacy Study of Acyclovir Against Cyprinid Herpesvirus 3 in Cyprinus carpio. Frontiers in Veterinary Science.

[71] Qureshi S.A. (1996). Function of Stat2 Protein in Transcriptional Activation by Alpha Interferon. Molecular and Cellular Biology.

[72] Rothenburg S., Brennan G. (2020). Species-Specific Host–Virus Interactions: Implications for Viral Host Range and Virulence. Trends in Microbiology.

[73] Ruben S.M. (1992). I-Rel: a novel rel-related protein that inhibits NF-kappa B transcriptional activity. Genes & Development.

[74] Seth R.B. (2005). Identification and Characterization of MAVS, a Mitochondrial Antiviral Signaling Protein that Activates NF-κB and IRF3. Cell.

[75] Shen N. (2022). High Expression of COL10A1 Is an Independent Predictive Poor Prognostic Biomarker and Associated with Immune Infiltration in Advanced Gastric Cancer Microenvironment. *Journal of Oncology*. Edited by Z. Shi.

[76] Sherman B.T. (2022). DAVID: a web server for functional enrichment analysis and functional annotation of gene lists (2021 update). Nucleic Acids Research.

[77] Smith N.C., Rise M.L., Christian S.L. (2019). A Comparison of the Innate and Adaptive Immune Systems in Cartilaginous Fish, Ray-Finned Fish, and Lobe-Finned Fish. Frontiers in Immunology.

[78] Sun R.-H. (2022). Blood brain barrier permeability and immune function of brain in rainbow trout responding to IHNV infection. Developmental & Comparative Immunology.

[79] Thoresen D.T. (2023). A rapid RIG-I signaling relay mediates efficient antiviral response. Molecular Cell.

[80] Thukral V. (2018). s8ORF2 protein of infectious salmon anaemia virus is a RNA-silencing suppressor and interacts with Salmon salar Mov10 (SsMov10) of the host RNAi machinery. Virus Genes.

[81] Troszok A. (2018). Acyclovir inhibits Cyprinid herpesvirus 3 multiplication in vitro. Journal of Fish Diseases.

[82] Troszok A., Roszko M. (2023). Thyme essential oil inhibits intracellular replication of CyHV -3 and inactivates extracellular virus. An in vitro study. Journal of Fish Diseases.

[83] Valero Y. (2015). Characterization of the IFN pathway in the teleost fish gonad against vertically transmitted viral nervous necrosis virus. Journal of General Virology.

[84] Wang L. (2021). MOV10 Helicase Interacts with Coronavirus Nucleocapsid Protein and Has Antiviral Activity. *mBio*. Edited by A.P. Geballe and S.I. Miller.

[85] Wang W. (2015). TRAF Family Member-associated NF-κB Activator (TANK) Inhibits Genotoxic Nuclear Factor κB Activation by Facilitating Deubiquitinase USP10-dependent Deubiquitination of TRAF6 Ligase. The Journal of Biological Chemistry.

[86] Wertz I.E. (2004). De-ubiquitination and ubiquitin ligase domains of A20 downregulate NF-κB signalling. Nature.

[87] Wickham H. (2016).

[88] Wickham H. (2019). Welcome to the Tidyverse. Journal of Open Source Software.

[89] WOAH (2022). https://www.woah.org/fileadmin/Home/eng/Health_standards/aahm/current/2.3.06_KHV.pdf.

[90] Xu P. (2014). Genome sequence and genetic diversity of the common carp, Cyprinus carpio. Nature Genetics.

[91] Xu P. (2019). The allotetraploid origin and asymmetrical genome evolution of the common carp Cyprinus carpio. Nature Communications.

[92] Xu Y. (2019). Development of two brain cell lines from goldfish and silver crucian carp and viral susceptibility to Cyprinid herpesivirus-2. In Vitro Cellular & Developmental Biology - Animal.

[93] Yan N., Chen Z.J. (2012). Intrinsic antiviral immunity. Nature Immunology.

[94] Yu F.-F. (2010). Fish virus-induced interferon exerts antiviral function through Stat1 pathway. Molecular Immunology.

[95] Zhang G. (2018). A proline deletion in IFNAR1 impairs IFN-signaling and underlies increased resistance to tuberculosis in humans. Nature Communications.

[96] Zhang S.-Y. (2007). TLR3 Deficiency in Patients with Herpes Simplex Encephalitis. Science.

[97] Zhu Y. (2019). Molecular characterization and functional analysis of interferon regulatory factor 9 (*irf9*) in common carp *Cyprinuscarpio* : a pivotal molecule in the Ifn response against pathogens. Journal of Fish Biology.

