# Supplementary material for: Differential transcriptomic responses in carp cell lines following activation of antiviral immune responses by poly I:C
Source: Comp Immunol Rep. 2026 Jun 29;11:200297. doi: 10.1016/j.cirep.2026.200297 (PMC13355688; doi:10.1016/j.cirep.2026.200297)
Supplement: Supplementary file 1 [file mmc1.docx]

Appendix 1: FeatureCounts code utilised in the analysis of RNA-seq data.

featureCounts -p -B -C -a /uoa/home/r01ls21/sharedscratch/genomes/Cyprinus_carpio_carpio.Cypcar_WagV4.0.110.gtf \

-o /uoa/home/r01ls21/sharedscratch/RNAseq_analysis/subread/C_24_0_4.txt \

/uoa/scratch/users/r01ls21/RNAseq_analysis/result/star_rsem/C_24_0_4.markdup.sorted.bam

featureCounts -p -B -C -a /uoa/home/r01ls21/sharedscratch/genomes/Cyprinus_carpio_carpio.Cypcar_WagV4.0.110.gtf \

-o /uoa/home/r01ls21/sharedscratch/RNAseq_analysis/subread/C_24_0_5.txt \

/uoa/scratch/users/r01ls21/RNAseq_analysis/result/star_rsem/C_24_0_5.markdup.sorted.bam

featureCounts -p -B -C -a /uoa/home/r01ls21/sharedscratch/genomes/Cyprinus_carpio_carpio.Cypcar_WagV4.0.110.gtf \

-o /uoa/home/r01ls21/sharedscratch/RNAseq_analysis/subread/C_24_0_6.txt \

/uoa/scratch/users/r01ls21/RNAseq_analysis/result/star_rsem/C_24_0_6.markdup.sorted.bam

featureCounts -p -B -C -a /uoa/home/r01ls21/sharedscratch/genomes/Cyprinus_carpio_carpio.Cypcar_WagV4.0.110.gtf \

-o /uoa/home/r01ls21/sharedscratch/RNAseq_analysis/subread/C_24_100_4.txt \

/uoa/scratch/users/r01ls21/RNAseq_analysis/result/star_rsem/C_24_100_4.markdup.sorted.bam

featureCounts -p -B -C -a /uoa/home/r01ls21/sharedscratch/genomes/Cyprinus_carpio_carpio.Cypcar_WagV4.0.110.gtf \

-o /uoa/home/r01ls21/sharedscratch/RNAseq_analysis/subread/C_24_100_5.txt \

/uoa/scratch/users/r01ls21/RNAseq_analysis/result/star_rsem/C_24_100_5.markdup.sorted.bam

featureCounts -p -B -C -a /uoa/home/r01ls21/sharedscratch/genomes/Cyprinus_carpio_carpio.Cypcar_WagV4.0.110.gtf \

-o /uoa/home/r01ls21/sharedscratch/RNAseq_analysis/subread/C_24_100_6.txt \

/uoa/scratch/users/r01ls21/RNAseq_analysis/result/star_rsem/C_24_100_6.markdup.sorted.bam

featureCounts -p -B -C -a /uoa/home/r01ls21/sharedscratch/genomes/Cyprinus_carpio_carpio.Cypcar_WagV4.0.110.gtf \

-o /uoa/home/r01ls21/sharedscratch/RNAseq_analysis/subread/K_24_0_4.txt \

/uoa/scratch/users/r01ls21/RNAseq_analysis/result/star_rsem/K_24_0_4.markdup.sorted.bam

featureCounts -p -B -C -a /uoa/home/r01ls21/sharedscratch/genomes/Cyprinus_carpio_carpio.Cypcar_WagV4.0.110.gtf \

-o /uoa/home/r01ls21/sharedscratch/RNAseq_analysis/subread/K_24_0_5.txt \

/uoa/scratch/users/r01ls21/RNAseq_analysis/result/star_rsem/K_24_0_5.markdup.sorted.bam

featureCounts -p -B -C -a /uoa/home/r01ls21/sharedscratch/genomes/Cyprinus_carpio_carpio.Cypcar_WagV4.0.110.gtf \

-o /uoa/home/r01ls21/sharedscratch/RNAseq_analysis/subread/K_24_0_6.txt \

/uoa/scratch/users/r01ls21/RNAseq_analysis/result/star_rsem/K_24_0_6.markdup.sorted.bam

featureCounts -p -B -C -a /uoa/home/r01ls21/sharedscratch/genomes/Cyprinus_carpio_carpio.Cypcar_WagV4.0.110.gtf \

-o /uoa/home/r01ls21/sharedscratch/RNAseq_analysis/subread/K_24_100_4.txt \

/uoa/scratch/users/r01ls21/RNAseq_analysis/result/star_rsem/K_24_100_4.markdup.sorted.bam

featureCounts -p -B -C -a /uoa/home/r01ls21/sharedscratch/genomes/Cyprinus_carpio_carpio.Cypcar_WagV4.0.110.gtf \

-o /uoa/home/r01ls21/sharedscratch/RNAseq_analysis/subread/K_24_100_5.txt \

/uoa/scratch/users/r01ls21/RNAseq_analysis/result/star_rsem/K_24_100_5.markdup.sorted.bam

featureCounts -p -B -C -a /uoa/home/r01ls21/sharedscratch/genomes/Cyprinus_carpio_carpio.Cypcar_WagV4.0.110.gtf \

-o /uoa/home/r01ls21/sharedscratch/RNAseq_analysis/subread/K_24_100_6.txt \

/uoa/scratch/users/r01ls21/RNAseq_analysis/result/star_rsem/K_24_100_6.markdup.sorted.bam

Table 1: Raw total and trimmed total sequences from all samples submitted to Novogene.

| Cell line | Treatment | Sample # | Raw total sequences | Trimmed total sequences |
| --- | --- | --- | --- | --- |
| CCB | Control | 1 | 35,615,885 | 35,597,080 |
| CCB | Control | 2 | 41,486,993 | 41,472,640 |
| CCB | Control | 3 | 36,086,090 | 36,075,006 |
| CCB | Poly I:C | 1 | 38,542,510 | 38,527,455 |
| CCB | Poly I:C | 2 | 31,067,854 | 31,055,414 |
| CCB | Poly I:C | 3 | 34,108,813 | 34,097,686 |
| KF1 | Control | 1 | 32,389,062 | 32,375,528 |
| KF1 | Control | 2 | 31,106,378 | 31,092,389 |
| KF1 | Control | 3 | 31,831,118 | 31,818,452 |
| KF1 | Poly I:C | 1 | 31,012,296 | 30,998,735 |
| KF1 | Poly I:C | 2 | 48,194,270 | 48,175,114 |
| KF1 | Poly I:C | 3 | 30,235,018 | 30,222,189 |

Table 2: Sequences and mapped sequences following processing using the STAR package in the nf-core pipeline.

| Cell line | Treatment | Sample # | % mapped | Total | Mapped |
| --- | --- | --- | --- | --- | --- |
| CCB | Control | 1 | 95.1 | 24,828,761 | 23,603,906 |
| CCB | Control | 2 | 89.2 | 28,996,496 | 25,860,872 |
| CCB | Control | 3 | 87.3 | 25,396,878 | 22,182,036 |
| CCB | Poly I:C | 1 | 90.2 | 27,744,534 | 25,038,777 |
| CCB | Poly I:C | 2 | 87.9 | 22,065,473 | 19,402,552 |
| CCB | Poly I:C | 3 | 89.7 | 24,202,361 | 21,717,689 |
| KF1 | Control | 1 | 87.7 | 21,940,124 | 19,241,234 |
| KF1 | Control | 2 | 77.5 | 20,729,055 | 16,067,447 |
| KF1 | Control | 3 | 87.2 | 21,952,543 | 19,141,588 |
| KF1 | Poly I:C | 1 | 80.7 | 21,801,428 | 17,602,285 |
| KF1 | Poly I:C | 2 | 84.3 | 34,177,477 | 28,801,606 |
| KF1 | Poly I:C | 3 | 83.6 | 21,242,006 | 17,751,580 |

Table 3: Percentages of assigned alignments from featureCounts. Unassigned alignments comprises all categories of nonassigned alignments including unmapped, singleton, and multimapped.

| Cell line | Treatment | Sample # | % assigned | % unassigned |
| --- | --- | --- | --- | --- |
| CCB | Control | 1 | 39.90 | 60.10 |
| CCB | Control | 2 | 37.79 | 62.21 |
| CCB | Control | 3 | 37.16 | 62.84 |
| CCB | Poly I:C | 1 | 38.41 | 61.59 |
| CCB | Poly I:C | 2 | 37.80 | 62.20 |
| CCB | Poly I:C | 3 | 38.28 | 61.72 |
| KF1 | Control | 1 | 38.02 | 61.98 |
| KF1 | Control | 2 | 34.30 | 65.70 |
| KF1 | Control | 3 | 37.64 | 62.36 |
| KF1 | Poly I:C | 1 | 36.11 | 63.89 |
| KF1 | Poly I:C | 2 | 32.20 | 67.80 |
| KF1 | Poly I:C | 3 | 36.46 | 63.54 |

Table 4: Top 20 significantly upregulated genes with a corresponding HGNC ID within CCB cells in response to poly I:C stimulation over 24 hours. (* = genes also found in the top 20 significantly upregulated genes in KF1 (Table 9))

| Ensembl Gene ID | Log_2_ Fold Change | HGNC ID | HGNC description | Subgenome |
| --- | --- | --- | --- | --- |
| ENSCCRG00000058912 | 10.69 | HSPB7 | heat shock protein family B7 | B |
| ENSCCRG00000077198* | 10.49 | ISG15 | ISG15 ubiquitin like modifier | B |
| ENSCCRG00000078547* | 10.12 | SAA1 | serum amyloid A1 | B |
| ENSCCRG00000007334* | 9.75 | LTA | lymphotoxin alpha | B |
| ENSCCRG00000058239* | 9.66 | IFIT1B | interferon induced protein with tetratricopeptide repeats 1B | B |
| ENSCCRG00000082645 | 9.36 | ISG15 | ISG15 ubiquitin like modifier | B |
| ENSCCRG00000036655 | 9.07 | CHST15 | carbohydrate sulfotransferase 15 | A |
| ENSCCRG00000064091 | 9.03 | WAS | WASP actin nucleation promoting factor | A |
| ENSCCRG00000038342* | 8.86 | MMP3 | matrix metallopeptidase 3 | A |
| ENSCCRG00000068371 | 8.77 | HELZ2 | helicase with zinc finger 2 | B |
| ENSCCRG00000066261 | 8.20 | URGCP | upregulator of cell proliferation | B |
| ENSCCRG00000063320 | 8.16 | TRIM28 | tripartite motif containing 28 | B |
| ENSCCRG00000064305* | 8.07 | RTP2 | receptor transporter protein 2 | A |
| ENSCCRG00000019384 | 8.05 | TRIM28 | tripartite motif containing 28 | B |
| ENSCCRG00000067473 | 8.02 | ZNFX1 | zinc finger NFX1-type containing 1 | B |
| ENSCCRG00000055972 | 7.98 | HELZ2 | helicase with zinc finger 2 | B |
| ENSCCRG00000064952 | 7.91 | IFI44L | interferon induced protein 44 like | B |
| ENSCCRG00000076968 | 7.86 | HS3ST3B1 | heparan sulfate-glucosamine 3-sulfotransferase 3B1 | B |
| ENSCCRG00000055283 | 7.79 | CNR2 | cannabinoid receptor 2 | B |
| ENSCCRG00000059078 | 7.33 | NCOA7 | nuclear receptor coactivator 7 | B |

Table 5: Top 20 significantly downregulated genes with a corresponding HGNC ID within CCB cells in response to poly I:C stimulation over 24 hours.

| Ensembl Gene ID | Log_2_ Fold Change | HGNC ID | HGNC description | Subgenome |
| --- | --- | --- | --- | --- |
| ENSCCRG00000078854 | -6.71 | EDIL3 | EGF like repeats and discoidin domains 3 | A |
| ENSCCRG00000044117 | -6.22 | RBMX2 | RNA binding motif protein X-linked 2 | A |
| ENSCCRG00000071402 | -6.14 | PTH2R | parathyroid hormone 2 receptor | B |
| ENSCCRG00000006431 | -5.99 | MME | membrane metalloendopeptidase | B |
| ENSCCRG00000055845 | -5.92 | BRINP2 | BMP/retinoic acid inducible neural specific 2 | B |
| ENSCCRG00000044104 | -5.34 | CPEB1 | cytoplasmic polyadenylation element binding protein 1 | A |
| ENSCCRG00000000514 | -5.27 | IRAG2 | inositol 1,4,5-triphosphate receptor associated 2 | A |
| ENSCCRG00000051946 | -5.27 | OPCML | opioid binding protein/cell adhesion molecule like | B |
| ENSCCRG00000013984 | -4.56 | DACH1 | dachshund family transcription factor 1 | B |
| ENSCCRG00000065253 | -4.45 | WFIKKN1 | WAP, follistatin/kazal, immunoglobulin, kunitz and netrin domain containing 1 | B |
| ENSCCRG00000033209 | -4.24 | ANGPTL4 | angiopoietin like 4 | A |
| ENSCCRG00000064787 | -3.96 | RBPMS2 | RNA binding protein, mRNA processing factor 2 | A |
| ENSCCRG00000050534 | -3.88 | MYO15B | myosin XVB | B |
| ENSCCRG00000043882 | -3.86 | SLC25A38 | solute carrier family 25 member 38 | B |
| ENSCCRG00000029318 | -3.83 | PIGS | phosphatidylinositol glycan anchor biosynthesis class S | A |
| ENSCCRG00000042850 | -3.81 | SYT11 | synaptotagmin 11 | B |
| ENSCCRG00000045528 | -3.77 | SLCO2A1 | solute carrier organic anion transporter family member 2A1 | B |
| ENSCCRG00000067128 | -3.76 | SLC35F3 | solute carrier family 35 member F3 | A |
| ENSCCRG00000034671 | -3.68 | NPR3 | natriuretic peptide receptor 3 | A |
| ENSCCRG00000050909 | -3.68 | FAM149A | family with sequence similarity 149 member A | B |

Table 6: Top 20 significantly upregulated genes with a corresponding HGNC ID within KF1 cells in response to poly I:C stimulation over 24 hours. (* = genes also found in the top 20 significantly upregulated genes in CCB (Table 7))

| Ensembl Gene ID | Log_2_ Fold Change | HGNC ID | HGNC description | Subgenome |
| --- | --- | --- | --- | --- |
| ENSCCRG00000007334* | 6.37 | LTA | lymphotoxin alpha | B |
| ENSCCRG00000078547* | 4.99 | SAA1 | serum amyloid A1 | B |
| ENSCCRG00000017142 | 4.79 | MMP11 | matrix metallopeptidase 11 | B |
| ENSCCRG00000077198* | 4.35 | ISG15 | ISG15 ubiquitin like modifier | B |
| ENSCCRG00000046494 | 4.06 | STEAP4 | STEAP4 metalloreductase | B |
| ENSCCRG00000061473 | 4.02 | CXCL8 | C-X-C motif chemokine ligand 8 | A |
| ENSCCRG00000027829 | 3.73 | MMP9 | matrix metallopeptidase 9 | B |
| ENSCCRG00000038342* | 3.60 | MMP3 | matrix metallopeptidase 3 | A |
| ENSCCRG00000058239* | 3.02 | IFIT1B | interferon induced protein with tetratricopeptide repeats 1B | B |
| ENSCCRG00000002099 | 3.01 | RSAD2 | radical S-adenosyl methionine domain containing 2 | B |
| ENSCCRG00000045543 | 2.90 | MOV10 | Mov10 RNA helicase | A |
| ENSCCRG00000081112 | 2.78 | HELZ2 | helicase with zinc finger 2 | B |
| ENSCCRG00000017422 | 2.68 | IFIT1B | interferon induced protein with tetratricopeptide repeats 1B | A |
| ENSCCRG00000073912 | 2.65 | IFIT1B | interferon induced protein with tetratricopeptide repeats 1B | B |
| ENSCCRG00000055315 | 2.52 | CMPK2 | cytidine/uridine monophosphate kinase 2 | A |
| ENSCCRG00000044075 | 2.29 | ACKR4 | atypical chemokine receptor 4 | A |
| ENSCCRG00000058577 | 2.29 | TNIP2 | TNFAIP3 interacting protein 2 | A |
| ENSCCRG00000040552 | 2.28 | ITGA2B | integrin subunit alpha 2b | B |
| ENSCCRG00000064305* | 2.27 | RTP2 | receptor transporter protein 2 | A |
| ENSCCRG00000052879 | 2.16 | IGFBP5 | insulin like growth factor binding protein 5 | B |

Table 7: Top 20 significantly downregulated genes with a corresponding HGNC ID within KF1 cells in response to poly I:C stimulation over 24 hours.

| Ensembl Gene ID | Log Fold Change | HGNC ID | HGNC description | Subgenome |
| --- | --- | --- | --- | --- |
| ENSCCRG00000056631 | -1.55 | ATOH8 | atonal bHLH transcription factor 8 | A |
| ENSCCRG00000076975 | -1.52 | PLTP | phospholipid transfer protein | B |
| ENSCCRG00000045455 | -1.46 | CHRNA2 | cholinergic receptor nicotinic alpha 2 subunit | B |
| ENSCCRG00000040722 | -1.40 | BGN | biglycan | B |
| ENSCCRG00000076105 | -1.40 | COL10A1 | collagen type X alpha 1 chain | A |
| ENSCCRG00000003092 | -1.28 | MYOC | myocilin | B |
| ENSCCRG00000079262 | -1.27 | C18orf21 | chromosome 18 open reading frame 21 | A |
| ENSCCRG00000003584 | -1.26 | COL11A2 | collagen type XI alpha 2 chain | B |
| ENSCCRG00000074688 | -1.23 | COL10A1 | collagen type X alpha 1 chain | B |
| ENSCCRG00000023967 | -1.15 | PHEX | phosphate regulating endopeptidase X-linked | A |
| ENSCCRG00000079131 | -1.13 | COL10A1 | collagen type X alpha 1 chain | B |
| ENSCCRG00000080192 | -1.12 | INSC | INSC spindle orientation adaptor protein | A |
| ENSCCRG00000082308 | -1.11 | COL10A1 | collagen type X alpha 1 chain | A |
| ENSCCRG00000011303 | -1.09 | GPT | glutamic--pyruvic transaminase | B |
| ENSCCRG00000054856 | -1.07 | PAH | phenylalanine hydroxylase | A |
| ENSCCRG00000024586 | -0.93 | CD82 | CD82 molecule | A |
| ENSCCRG00000018073 | -0.90 | SGCA | sarcoglycan alpha | A |
| ENSCCRG00000015937 | -0.88 | SH3GL3 | SH3 domain containing GRB2 like 3, endophilin A3 | A |
| ENSCCRG00000074936 | -0.88 | SLC20A1 | solute carrier family 20 member 1 | A |
| ENSCCRG00000062845 | -0.86 | DLX2 | distal-less homeobox 2 | A |

Table 8: Upregulated genes associated with the GO term “defence response to virus” (GO:0051607) in CCB & KF1 following analysis by the DAVID suite. (Blank spaces denote genes where significant expression (padj < 0.05, LF2C > 1) was not found).

| HGNC ID | Ensembl Gene ID | Log_2_ Fold Change (CCB) | Log2 Fold Change (KF1) | Subgenome |
| --- | --- | --- | --- | --- |
| CXCL9 | ENSCCRG00000057118 | 4.81 |  | B |
| NLRC5 | ENSCCRG00000037228 | 4.10 |  | B |
| NLRC5 | ENSCCRG00000014861 | 2.29 |  | A |
| ADAR | ENSCCRG00000039665 | 1.19 |  | A |
| TANK | ENSCCRG00000039972 | 2.11 | 1.12 | A |
| TANK | ENSCCRG00000039987 | 1.92 | 1.11 | B |
| IFI44L | ENSCCRG00000064952 | 7.91 |  | B |
| IFI44L | ENSCCRG00000070613 | 5.00 |  | B |
| IFI44L | ENSCCRG00000019301 | 4.35 |  | B |
| IFI44L | ENSCCRG00000004924 | 3.94 |  | A |
| IFI44L | ENSCCRG00000007709 | 2.19 |  | A |
| IFIH1 | ENSCCRG00000041831 | 2.49 |  | A |
| IFIH1 | ENSCCRG00000013858 | 2.16 |  | B |
| DHX58 | ENSCCRG00000033653 | 5.92 |  | A |
| DHX58 | ENSCCRG00000077125 | 2.99 |  | - |
| DHX58 | ENSCCRG00000065109 | 2.95 |  | B |
| DHX58 | ENSCCRG00000066329 | 1.79 |  | B |
| GBP1 | ENSCCRG00000051785 | 1.09 |  | B |
| MLKL | ENSCCRG00000046262 | 1.07 |  | B |
| DTX3L | ENSCCRG00000018170 | 4.93 |  | A |
| DTX3L | ENSCCRG00000058949 | 1.94 |  | B |
| TREX1 | ENSCCRG00000067383 | 2.86 |  | B |
| STAT2 | ENSCCRG00000049842 | 3.28 |  | B |
| EIF2AK2 | ENSCCRG00000012933 | 2.07 |  | B |
| ISG15 | ENSCCRG00000077198 | 10.49 | 4.35 | B |
| ISG15 | ENSCCRG00000082645 | 9.36 |  | B |
| ISG15 | ENSCCRG00000062500 | 5.62 | 1.96 | A |
| ISG15 | ENSCCRG00000060054 | 4.51 | 1.89 | B |
| ISG15 | ENSCCRG00000048369 | 2.31 | 1.91 | B |
| ISG15 | ENSCCRG00000035128 | 1.64 |  | A |
| ISG20 | ENSCCRG00000049015 | 1.59 |  | B |
| MOV10 | ENSCCRG00000045543 | 5.76 | 2.9 | A |
| MOV10 | ENSCCRG00000058348 | 1.41 |  | A |
| MOV10 | ENSCCRG00000015515 | 1.16 |  | B |
| ZNFX1 | ENSCCRG00000067473 | 8.02 | 1.56 | B |
| ZNFX1 | ENSCCRG00000067854 | 1.84 |  | A |
| IRF3 | ENSCCRG00000030888 | 4.31 |  | B |
| IRF3 | ENSCCRG00000025004 | 2.33 |  | A |
| IFI27 | ENSCCRG00000076856 | 1.38 |  | B |
| IRF1 | ENSCCRG00000010768 | 5.23 |  | B |
| IRF1 | ENSCCRG00000037300 | 1.32 |  | A |
| CD207 | ENSCCRG00000003170 | 1.06 |  | A |
| IFIT1B | ENSCCRG00000058239 | 9.66 | 3.02 | B |
| IFIT1B | ENSCCRG00000073912 | 6.18 | 2.65 | B |
| IFIT1B | ENSCCRG00000017422 | 5.65 | 2.68 | A |
| IRF7 | ENSCCRG00000029695 | 5.30 |  | B |
| SHFL | ENSCCRG00000016382 | 1.05 |  | A |
| IRF9 | ENSCCRG00000047792 | 1.09 |  | B |
| RIGI | ENSCCRG00000057075 | 2.80 |  | A |
| RSAD2 | ENSCCRG00000002099 |  | 3.01 | B |

Table 9: KEGG pathway terms associated with HGNC-annotated upregulated genes in CCB cells following 24h of stimulation with 100µg/ml of poly I:C.

| KEGG pathway term | Fold Enrichment | | Gene Count | p-value |
| --- | --- | --- | --- | --- |
| RIG-I-like receptor signaling pathway | | 6.9 | 12 | 2.79X10^-4^ |
| Measles | | 4.5 | 15 | 8.13X10^-4^ |
| TNF signaling pathway | | 4.5 | 13 | 2.03X10^-3^ |
| Epstein-Barr virus infection | | 3.5 | 17 | 2.03X10^-3^ |
| Human papillomavirus infection | | 2.7 | 22 | 2.75X10^-3^ |
| Osteoclast differentiation | | 3.8 | 13 | 6.74X10^-3^ |
| Pathways in cancer | | 2.2 | 28 | 6.74X10^-3^ |
| Coronavirus disease - COVID-19 | | 3.0 | 17 | 6.74X10^-3^ |
| Hepatitis C | | 3.4 | 13 | 1.35X10^-2^ |
| Hepatitis B | | 3.3 | 13 | 1.52X10^-2^ |
| Influenza A | | 3.1 | 13 | 2.23X10^-2^ |
| Oxytocin signaling pathway | | 3.2 | 12 | 2.70X10^-2^ |
| Proteoglycans in cancer | | 2.8 | 14 | 2.70X10^-2^ |
| Cell adhesion molecules | | 3.2 | 12 | 2.76X10^-2^ |
| Necroptosis | | 3.1 | 12 | 2.79X10^-2^ |
| Th1 and Th2 cell differentiation | | 4.0 | 9 | 2.79X10^-2^ |
| NOD-like receptor signaling pathway | | 2.8 | 13 | 3.25X10^-2^ |
| Kaposi sarcoma-associated herpesvirus infection | | 2.7 | 13 | 4.17X10^-2^ |


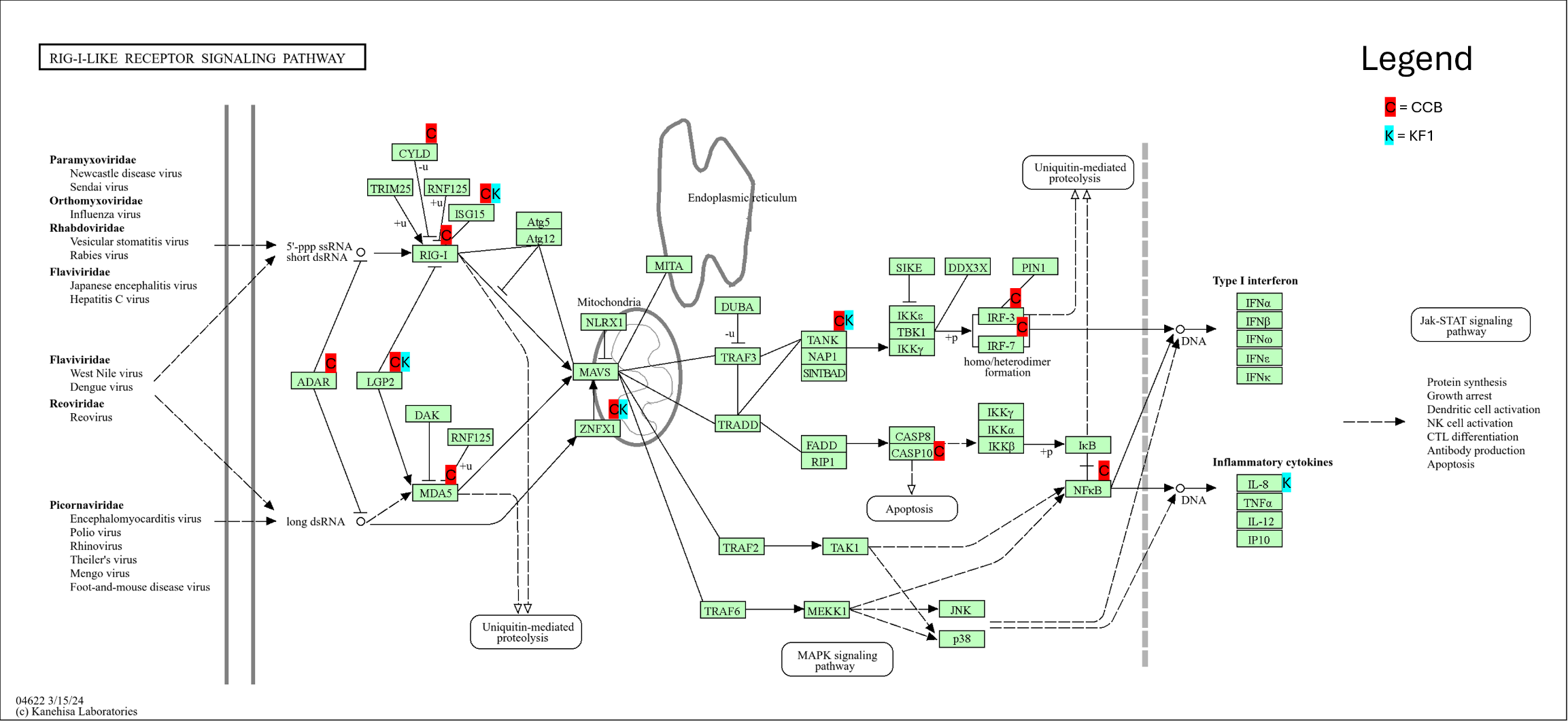


Figure 1: A KEGG pathway diagram of the RIG-I-like receptor signalling pathway in HGNC-annotated upregulated genes in CCB (p = 2.8x10^-4^, 6.8-fold) and KF1 (p = 3.9x10^-3^, 23.6-fold). Solid arrows represent known molecular interaction or relation, while dashed lines represent indirect links or unknown reactions. A line ending in a flat line denotes inhibition. The letters denoting +u represent ubiquitination in protein-protein interactions, while +p denotes phosphorylation.
